# Understanding pressure excursions in extracorporeal technologies: The role of shear stress and hematologic predisposition

**DOI:** 10.1051/ject/2026003

**Published:** 2026-06-19

**Authors:** Youssef El Dsouki, Chiara Cirullo, Assunta D’Alessandro, Ignazio Condello

**Affiliations:** 1 Faculty of Health, Medicine and Life Sciences, Cardiovascular Research Institute Maastricht (CARIM), Maastricht University The Netherlands; 2 AAST Sette Laghi 21100 Varese VA Italy; 3 School of Medicine and Surgery, University of Insubria Via Ravasi, 2 21100 Varese VA Italy

*Dear Editor,*

Platelets play a pivotal role in hemostasis, where their activation is tightly regulated by biochemical agonists such as thrombin, collagen, and adenosine diphosphate, all of which signal through calcium-mediated intracellular pathways. Under physiological conditions, this tightly controlled system ensures a rapid and localized response to vascular injury. During extracorporeal circulation, such as cardiopulmonary bypass (CPB) and extracorporeal membrane oxygenation (ECMO), platelets are instead exposed to artificial surfaces and abnormal flow dynamics. These artificial conditions introduce non-physiological forces, including elevated shear stress and turbulence, particularly across the oxygenator, which represents one of the most flow-restrictive and biologically active components of the circuit. Shear stress has long been recognized as a potent mechanical activator of platelets, capable of triggering intracellular calcium influx even in the absence of endothelial injury. This observation suggests that platelets circulating within CPB and ECMO circuits may become inappropriately activated purely as a function of circuit mechanics. Once activated, platelets may adhere to synthetic surfaces and form microaggregates, initiating a cascade of events that progressively impair oxygenator performance. As resistance within the oxygenator increases, transmembrane pressure rises, potentially culminating in a clinically relevant adverse event known as high-pressure excursion (HPE). HPE frequently necessitates oxygenator replacement, an intervention that carries procedural risk, interrupts support continuity, and increases both cost and circuit manipulation. Understanding the mechanisms underlying pressure excursions is therefore essential to improving extracorporeal support safety and durability.

1. Platelet structure and physiological role
Platelets are anucleate cytoplasmic fragments derived from megakaryocytes, with a typical diameter of 2–3 μm. Despite lacking nuclei, they are metabolically active and capable of protein synthesis through residual messenger RNA. Platelets contain a complex intracellular architecture consisting of a dynamic cytoskeleton, membrane receptors, granules, and signaling pathways that allow rapid response to vascular injury [1]. The platelet membrane expresses glycoprotein complexes essential for adhesion and aggregation, including the GPIb-IX-V complex, which mediates binding to von Willebrand factor on exposed subendothelial collagen, and GPIIb/IIIa, which facilitates fibrinogen-mediated platelet crosslinking [2]. Alpha granules store fibrinogen, von Willebrand factor, platelet factor 4, and P-selectin, while dense granules contain calcium, adenosine diphosphate, and serotonin. Under physiological conditions, platelet activation is tightly regulated by endothelial-derived inhibitory mediators such as nitric oxide and prostacyclin, which maintain circulating platelets in a quiescent state. This finely balanced regulatory system is profoundly altered in extracorporeal circulation, where platelets are removed from endothelial control and exposed to non-physiological mechanical forces.
2. Shear stress, platelet activation, and high-pressure excursion
Shear stress is a tangential mechanical force generated by blood flow along a surface. In physiological arterial circulation, shear stress typically ranges between 10 and 40 dyn/cm². Within extracorporeal circuits, however, localized shear stress can rise substantially, particularly in regions of flow restriction such as oxygenator fiber bundles. Platelet activation induced by shear stress differs fundamentally from classical receptor-mediated pathways [3]. Rather than relying on biochemical agonists, shear-mediated activation occurs primarily through mechanosensitive calcium channels, leading to non-physiological intracellular calcium influx. This calcium entry triggers early activation events, including cytoskeletal reorganization, phosphatidylserine exposure, and granule release. Importantly, this form of activation is not inhibited by standard systemic anticoagulation with unfractionated heparin, which targets thrombin and factor Xa but does not prevent mechanically induced platelet signaling [4]. Repeated or sustained exposure to elevated shear stress may result in platelet priming, a sub-threshold activation state characterized by heightened sensitivity to subsequent mechanical or biochemical stimuli. Primed platelets are more likely to adhere, aggregate, and propagate microthrombus formation, particularly when exposed to artificial surfaces. As microaggregates accumulate within the oxygenator, local flow resistance increases, further elevating shear stress and establishing a self-reinforcing cycle that can culminate in HPE.
3. The pathophysiology of pressure drop across the oxygenator
Oxygenators are the functional core of extracorporeal gas exchange. In contemporary hollow-fiber oxygenators, blood flows around densely packed polymethylpentene fibers, while oxygen and carbon dioxide diffuse across the fiber membranes. The fiber bundle represents a significant hydraulic resistance, which becomes increasingly relevant as blood viscosity rises or microaggregate formation occurs. The transmembrane pressure drop (ΔP) across the oxygenator is defined as the pressure difference between the inlet and outlet ports. Under normal conditions, ΔP remains stable at low values when flow is laminar, and fiber pathways are unobstructed [5]. However, platelet adhesion and microthrombus formation along the fiber surfaces progressively narrow flow channels, increasing local resistance. This obstruction leads to a progressive increase in pressure drop across the oxygenator [6]. As ΔP rises, local shear stress intensifies, generating regions of flow acceleration, recirculation, and turbulence. Experimental models and computational fluid dynamics studies have demonstrated that such conditions can produce shear stress values exceeding physiological thresholds, sufficient to induce further platelet activation and red blood cell damage [7]. This process exacerbates the initial obstruction and accelerates deterioration of oxygenator performance (Figure 1).
  **Figure 1:**
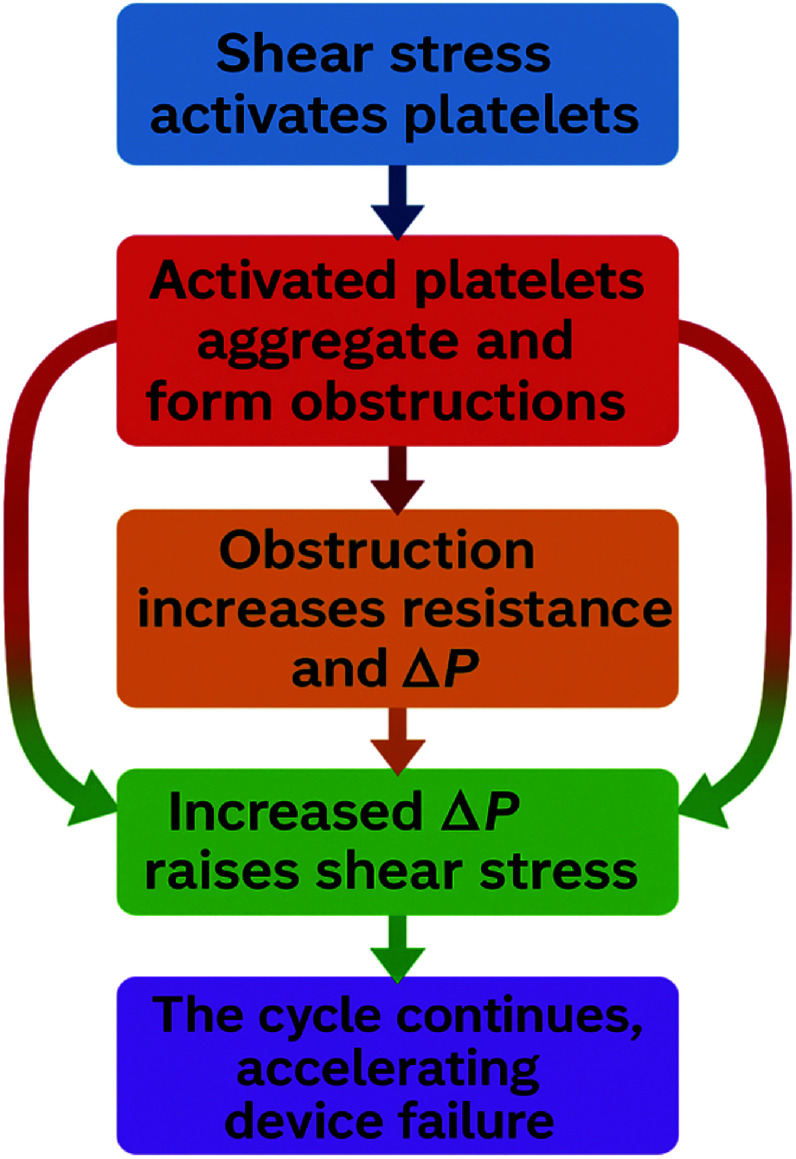
Mechanobiological loop underlying pressure excursions during extracorporeal circulation.
  This sequence establishes a self-reinforcing mechanobiological loop: (Figure 1)
  This flowchart illustrates the proposed pathophysiological cycle driving pressure excursions in oxygenators during CPB and ECMO. Mechanical shear stress (blue) initiates calcium-dependent platelet activation, independent of biochemical agonists. Activated platelets (red) adhere and aggregate on the oxygenator’s synthetic surfaces, forming microthrombi that progressively obstruct flow channels. These obstructions (orange) increase transmembrane pressure drop (ΔP), which further raises local shear stress (green), promoting additional platelet activation. The cycle continues (purple), accelerating oxygenator dysfunction and potentially necessitating early device replacement. Color coding reflects the temporal progression and functional impact of each phase within the self-reinforcing loop. The platelets represent a central but not exclusive component of a broader thrombo-mechanical process involving surface adhesion and microthrombus propagation.
4. Extracorporeal circuits and the loss of physiological regulation
In extracorporeal circulation systems, blood is exposed to synthetic materials such as polymethylpentene, polycarbonate, and silicone, which lack the antithrombotic and regulatory properties of native endothelium. Contact with these surfaces promotes adsorption of plasma proteins, complement activation, contact pathway triggering, and platelet adhesion [3]. While systemic anticoagulation with unfractionated heparin effectively suppresses thrombin generation and fibrin formation, it does not restore endothelial-mediated regulation of platelet function. Consequently, platelet activation driven by mechanical shear stress occurs in an environment devoid of nitric oxide, prostacyclin, and other physiological inhibitory signals [4]. This loss of regulatory control allows mechanically induced platelet activation to proceed unchecked, particularly within high-shear regions of the circuit. The combination of artificial surfaces, elevated shear stress, and impaired physiological regulation creates conditions favorable for progressive platelet activation, aggregation, and oxygenator dysfunction.
5. Future perspective
The relationship between mechanical stress and platelet activation in extracorporeal circuits is increasingly recognized as a critical determinant of oxygenator performance and failure. Under physiological conditions, platelets remain quiescent until activated by tightly regulated biochemical signals in response to vascular injury. During CPB and ECMO, however, platelets are exposed to synthetic surfaces and abnormal shear forces that can bypass receptor-mediated pathways and directly induce intracellular calcium signaling [5, 6]. This mechanically induced activation promotes platelet adhesion and microaggregate formation within the oxygenator fiber bundle. These early obstructions increase flow resistance and transmembrane pressure, further elevating shear stress and reinforcing a vicious mechanobiological cycle. Clinically, this process manifests as HPE, a warning sign of imminent oxygenator dysfunction that often necessitates urgent device replacement. Importantly, this phenomenon can occur despite adequate anticoagulation, highlighting a fundamental limitation of current extracorporeal management strategies. Hematologic factors such as elevated platelet count, increased hematocrit, and higher blood viscosity may further predispose certain patients to this complication [7]. While prostacyclin analogs such as epoprostenol have demonstrated safety in limited clinical series, their role in preventing shear-induced platelet activation requires further investigation [8]. Recognizing shear-mediated platelet activation as both a marker and mediator of circuit stress has important implications for clinical practice and device design. Future strategies should focus on reducing shear stress through improved oxygenator geometry, enhancing hemocompatibility with advanced surface coatings, implementing real-time pressure monitoring, and exploring targeted antiplatelet approaches aimed specifically at mechanically induced platelet trauma. Experimental validation of this proposed mechanobiological loop is essential to define activation thresholds and guide preventive interventions, with the ultimate goal of improving device longevity and patient outcomes in extracorporeal life support.

## Data Availability

The data supporting this study’s findings are available from the corresponding author upon reasonable request.
